# Comparative Mitogenomics of *Meiacanthus* Blennies Reveals Venom System Evolution and Adaptive Traits

**DOI:** 10.3390/ani16152315

**Published:** 2026-07-27

**Authors:** Tengda Luo, Haiyan Yu, Weilin Xiao, Boqiong Wu, Zixuan Liu, Yuqing Tang, Qiang Lin, Yanhong Zhang

**Affiliations:** 1 State Key Laboratory of Tropical Oceanography, Guangdong Provincial Key Laboratory of Applied Marine Biology, South China Sea Institute of Oceanology, Chinese Academy of Sciences, Guangzhou 510000, China; luotengda23@mails.ucas.ac.cn (T.L.); 33120220156705@stu.xmu.edu.cn (H.Y.); xiaoweilin21@mails.ucas.ac.cn (W.X.); wuboqiong24@mails.ucas.ac.cn (B.W.); liuzixuan232@mails.ucas.ac.cn (Z.L.); tangyuqing24@mails.ucas.ac.cn (Y.T.); 2 Institute of Ocean Eco-Environmental Engineering, Sanya 572000, China; 3 University of Chinese Academy of Sciences, Beijing 100049, China

**Keywords:** oral venom system, ancestral status reconstruction, phylogenetic analyses, positive selection, *Meiacanthus*

## Abstract

*Meiacanthus* is a genus of nemophine blennies with an oral venom system, which includes buccal venom glands and canine fangs, and its evolutionary origin remains poorly understood. Here, we sequenced complete mitochondrial genomes of *Meiacanthus* species and their closely related non-venomous species, and performed comparative genomic and phylogenetic analyses to explore *Meiacanthus*’ evolution. Phylogenetic reconstruction identified *Meiacanthus* as sister to the (*Aspidontus* + *Petroscirtes*) clade, and suggested a probable three-step evolutionary sequence for its oral venom system. Codon usage bias analysis revealed that both natural selection and nucleotide mutations influence nemophine codon usage, shaping a bias that likely plays a crucial role in translational efficiency and regulating mitochondrial gene function. We also identified a unique nemophine tRNA-Cys (GCA) with a degraded DHU loop, which is prompted to be associated with a corresponding shift in codon usage preference. Additionally, all mitochondrial protein-coding genes of *Meiacanthus* except *Cytb* exhibited positive selection, with *ND4* and *ND5* having positively selected sites, likely linked to elevated energy demands for toxin synthesis. Our findings provide the first mitogenomic evidence illuminating the evolutionary pathway of the oral venom system in *Meiacanthus*, offering crucial insights into the evolution of oral venom systems in non-tetrapod vertebrates.

## Simple Summary

*Meiacanthus* is a genus of blennid fishes, which are the only coral reef fishes known to possess an oral venom system. In this research, we trace the evolutionary history of *Meiacanthus* and reveal that *Meiacanthus* likely evolved canine fangs prior to oral venom glands, which is a sequence contrary to that in venomous snakes. Codon usage bias in the mitochondrial genome appears shaped by both mutation and natural selection, likely driven by selective pressures on the fang and venom gland complex. We also identified a structurally simplified tRNA-Cys with a degraded DHU loop, which results in a corresponding shift in codon usage preference. Furthermore, most mitochondrial genes exhibit positive selection, indicating elevated energy demands associated with venom production. These findings clarify the phylogenetic relationships and molecular evolution of this unique coral reef lineage and provide a valuable model for understanding the origin and evolution of vertebrate oral venom systems.

## 1. Introduction

*Meiacanthus*, a genus of fangblennies commonly known as venomous fangblennies, is one of the three fish lineages that possess the oral venom system [1], and is classified within the tribe Nemophini of the family Blenniidae. Among the fish confirmed to possess an oral venom system, *Meiacanthus* is the only euteleost and the only representative inhabiting coral reef ecosystems, holding a unique ecological niche in the habitat. *Meiacanthus* is distributed throughout the tropical Indo-Pacific, with two notable exceptions: *M. kamoharai* occurs in temperate waters, whereas *M. anema* inhabits brackish estuaries and even freshwater environments.

As a venomous fish, *Meiacanthus* possesses mandibular fangs associated with venom glands, forming a specialized morphological trait that serves as an effective defensive weapon against predators. The venom produced by *Meiacanthus* has been shown to cause at least hypotensive effects and weak neurotoxicity [2], providing an effective defensive mechanism against predators within the complex predator–prey interactions of coral reef ecosystems. This venom-based defense has contributed to the evolution of a complex mimicry system, in which *Meiacanthus* serves as a key venomous model within coral reef communities [3].

Over the past decades, *Meiacanthus* has piqued the interest of researchers from multiple perspectives, including ethology, toxicology, and phylogenetics. However, despite being recognized as venomous fish more than half a century ago [4,5,6], our knowledge of these fishes remains severely restricted, particularly at the genetic level. In terms of phylogenetic research, several studies have investigated the phylogeny of the family Blenniidae, but their inferences regarding the phylogenetic relationships among genera within Blenniidae are not consistent. Nevertheless, all of them support the monophyletic status of the tribe Nemophini, and consider this tribe to be sister to *Omobranchus* and *Enchelyurus*, forming a monophyletic group characterized by enlarged canine fangs. However, the phylogenetic relationship among nemophine genera is still under debate, and the phylogenetic position of *Meiacanthus* is also unresolved [7]. Previous studies based on different gene datasets have recovered conflicting sister-group relationships, placing *Meiacanthus* as sister to *Plagiotremus*, (*Plagiotremus* + *Xiphasia*), or (*Aspidontus* + *Petroscirtes*) [2,8,9,10]. These inconsistent findings underscore the need for further research to accurately determine the phylogenetic position of *Meiacanthus*, as this would significantly enhance our understanding of the evolution of their oral venom system.

When it comes to the evolutionary origin of the *Meiacanthus* venom system, existing studies are rather superficial. Most related research has mainly focused on the phylogeny of the entire Blenniidae family, with scant attention paid to the tribe Nemophini. It has been hypothesized that *Meiacanthus* first evolved enlarged fangs before acquiring venom glands, which is in contrast to the pattern proposed for snakes [2]. It has also been argued that the emergence of the buccal venom system of *Meiacanthus* was followed by rapid speciation [9]. Nevertheless, these studies neither delved into gene-level mechanisms nor extended their comparisons beyond Blenniidae. As a result, the evolutionary origin and diversification of the oral venom system in *Meiacanthus*, as well as its relationship to other fish lineages, remain poorly understood. This lack of in-depth knowledge not only limits our understanding of *Meiacanthus* itself but also hampers our exploration of the evolution of rare defensive mechanisms in fishes and non-tetrapod vertebrates in general.

To address the significant gaps in our understanding of *Meiacanthus*, this study undertakes a comprehensive analysis. We present the complete mitochondrial genomes of four *Meiacanthus* species and three non-venomous nemophine species. By employing a suite of advanced analytical methods, our research not only clarified the phylogenetic position of *Meiacanthus* blennies, but also shed light on the evolutionary history of this specialized venomous fish lineage as well as its oral venom system. By doing so, we hope to offer crucial insights into understanding the origin of venom in non-tetrapod vertebrates, thereby significantly advancing our knowledge of the evolution of this important survival strategy across the animal kingdom.

## 2. Materials and Methods

### 2.1. Materials

Specimens of four *Meiacanthus* species (*M. atrodorsalis*, *M. grammistes*, *M. mossambicus* and *M. tongaensis*) and three non-venomous species of the tribe Nemophini (*Aspidontus taeniatus*, *Plagiotremus rhinorhynchos* and *Xiphasia setifer*) were acquired from the commercial aquarium trade. For subsequent molecular analyses, muscle tissues were aseptically dissected from the fresh specimens. Dissected tissues were immediately snap-frozen in liquid nitrogen to preserve nucleic acid integrity, and were thereafter stored at −80 °C until genomic DNA extraction.

### 2.2. Acquisition of Mitochondrial Genome Sequences and Genome Annotation

Tissues were sequenced by GeneRed Co., Ltd. (Wuhan, China). DNA samples were randomly fragmented into 300–500 bp fragments using a Covaris ultrasonicator (Covaris, Woburn, MA, USA). A library was constructed and then sequenced using the Illumina NovaSeq platform with PE150 sequencing to obtain raw data (Illumina, San Diego, CA, USA). The raw reads were subsequently filtered to obtain clean data using SOAPnuke 2.1.7 software. The mitochondrial genomes were directly assembled from clean data through NOVOPlasty 4.3.1 [11]. The sequencing coverage depth of the mitochondrial genome is shown in Supporting Information Figure S1. The assembled mitochondrial genomes were annotated using MITOS 2.1.0 [12], and genome maps were generated with Proksee (https://proksee.ca, accessed on 21 July 2026) [13]. The secondary structures of tRNAs were predicted using tRNAscan-SE 2.0.9 [14] and visualized through VARNA v3-93 [15].

### 2.3. Phylogenetic Analysis and Ancestral State Reconstruction

The complete mitochondrial genomes and coding sequences (CDSs) of other species including *Petroscirtes breviceps* were downloaded from GenBank (www.ncbi.nlm.nih.gov/genbank/ (accessed on 21 July 2026)) for phylogenetic analysis. The GenBank accession numbers for these sequences are shown in Supporting Information Table S1. The dataset for phylogenetic analysis included 84 species, encompassing all available mitogenomes of blennid genera from GenBank, the venomous one-jaw eel *Monognathus jesperseni*, and several fang-bearing fish species. These sequences were analyzed together with mitochondrial genome data provided by this study. Prior to the phylogenetic analysis, thirteen protein-coding genes were aligned using MAFFT v7.526 [16] and trimmed with trimAl v1.4 [17]. The aligned genes were then concatenated for phylogenetic analysis. The best-fit substitution model (GTR + F + R9) was determined using ModelFinder in IQ-TREE v2.1.4-beta [18], and the GTRGAMMA model was ultimately selected for tree construction. The phylogenetic tree was inferred using RAxML v8.2.12 with 1000 bootstrap replicates [19].

To construct the time-calibrated phylogenetic tree, we set five fossil calibration points. *Tottoriblennius hiraoi*, a member of the tribe Nemophini that lived during the Middle to Late Miocene and was discovered in Japan, was used to calibrate the most recent common ancestor of Nemophini. Early fossil records of the crown groups of Blenniidae, Actinopterygii, Gnathostomata, and Vertebrata are extremely scarce, and no fossils have yet been found that can be confidently assigned near their origin nodes. Therefore, we adopted molecular phylogenetic study results and data from the Tree of Life website to set these divergence times at 65–60 Ma, 342.5–298.8 Ma, 495.2–440.8 Ma, and 652–550 Ma, respectively. The time-calibrated phylogenetic tree was constructed using mcmctree in PAML version 4.9j [20] and then visualized in FigTree v1.4.4. The ancestral state reconstruction was performed using the ace function in the R package ape 5.8.1 with the Equal Rates model [21].

### 2.4. Speciation Rate Analysis

To estimate speciation rates through time in the diversification of *Meiacanthus*, we obtained mitogenomic data from Blenniiformes available on GenBank and combined it with the mitogenome data generated in this study. The GenBank accession numbers for these sequences are shown in Supporting Information Table S2. Phylogenetic analysis was conducted using the GTRGAMMAI model in RAxML v8.2.12. Bootstrap analyses were performed with 1000 replicates, and GenBank accession numbers for the sequences are provided in Supplementary Table S2. The time-calibrated phylogenetic tree was constructed using mcmctree in PAML version 4.9j [20]. Diversification analyses were performed using this time-calibrated tree with BAMM v2.5.0. A speciation–extinction model was implemented, and Markov chain Monte Carlo (MCMC) sampling was run for 10,000,000 generations, with samples collected every 1000 generations. Four Metropolis-coupled MCMC chains were used. The prior expectation for the number of diversification rate shifts was set to 1.0, and species-specific sampling fractions were incorporated into the analysis. The sampling fraction for each lineage was set based on the ratio of the number of species included in the analysis to the total number of known species in each genus. The output files from BAMM were processed in R using the BAMMtools package v2.1.12 [22], and the first 10% of MCMC samples were discarded as burn-in. To visualize the distribution of speciation rates across the phylogeny, mean posterior speciation rates (λ) were mapped onto the tree using a polar coordinate layout, with branch colors representing the magnitude of the rates. Tip-specific speciation rates were extracted to define the range of the color scale. Temporal variation in speciation dynamics within the focal lineage was examined by defining the foreground lineage as the crown group *Meiacanthus*, and the background lineage as other species. The most recent common ancestor (MRCA) of *Meiacanthus* was identified and used to separate the foreground and background lineages.

### 2.5. Codon Usage Bias (CUB) Indices

Codon usage bias (CUB) refers to the differential usage frequency of the synonymous codons in the CDS. Analysis of CUB patterns and their influencing factors among species can facilitate the understanding of the molecular mechanism of biological adaptation to the environment [23]. The CUB indices for all protein-coding sequences across eight nemophine species were calculated using CodonW 1.4.2 and EMBOSS 6.6.0 [24].

#### 2.5.1. Nucleotide Composition

The GC content of the complete mitochondrial genomes, as well as the GC1s, the GC2s, and the GC3s contents (only complete codons considered) of each CDS, were determined with EMBOSS 6.6.0. The overall GC content of the CDSs, the GC content of each CDS, and the frequencies of each mononucleotide at the third codon position (A3, C3, T3, and G3) were obtained using CodonW 1.4.2.

#### 2.5.2. Relative Synonymous Codon Usage (RSCU)

The RSCU value of a codon is calculated as the ratio of its observed frequency to the expected frequency (the mean frequency of all synonymous codons for the corresponding amino acid) [25], and the RFSC value of a codon is calculated as the proportion of its occurrences among all synonymous codons encoding the same amino acid. In this research, we determined the RSCU value of each mitochondrial genome sequence using EMBOSS 6.6.0, and calculated the RFSC value via the formula below:

(1) $${RFSC}_{ij}=\frac{{RSCU}_{ij}}{m_{j}}$$

where m_j_ represents the total number of synonymous codons that encode the jth amino acid, and RSCU_ij_ represents the RSCU value for the ith codon of the jth amino acid.

#### 2.5.3. Codon Adaptation Index (CAI) Analysis

CAI is the degree of coincidence between synonymous codons in the coding region and optimal codon frequency, with values ranging from 0 to 1. For a gene, the higher the CAI value is, the stronger the adaptability is. CAI is therefore widely applied in the evaluation of gene expression. CAI values are calculated using the following formula [26]:

(2) $$w_{\mathrm{ij}}=\frac{{\mathrm{RSCU}}_{\mathrm{ig}}}{{\mathrm{RSCU}}_{\mathrm{imax}}}=\frac{x_{\mathrm{ij}}}{x_{\mathrm{imax}}}$$

(3) $$\mathrm{CAI}={\left(\prod_{K=1}^{L}W_{K}\right)}^{\frac{1}{L}}$$

where x_ij_ denotes the frequency of the ith codon for the jth amino acid, RSCU_imax_ and x_imax_ represent the RSCU value and x-value of the most frequently used codon for the ith amino acid, respectively, and L is the total number of codons in the gene. In this study, CAI values were computed using CodonW 1.4.2.

#### 2.5.4. Codon Bias Index (CBI) Analysis

The CBI value reflects the extent to which a gene preferentially uses highly represented codons. This index is commonly applied to predict gene expression levels in coding sequences and to measure directional codon bias, indicating the degree to which a gene utilizes a subset of optimal codons. The formula of CBI is as follows [26]:

(4) $$\begin{array}{c} CBI\;=\;\frac{N_{\mathrm{opt}}-N_{\mathrm{ran}}}{N_{\mathrm{tot}}-N_{\mathrm{ran}}} \end{array}$$

where N_opt_ is the total number of optimal codons observed in the gene, N_ran_ represents the expected number of optimal codons under random synonymous codon usage for the same amino acid sequence, and N_tot_ is the total number of occurrences of the amino acid encoded by the optimal codons in the gene. In this study, CAI values were computed using CodonW 1.4.2. The r-value and *p*-value between CAI and CBI were calculated using Python 3.13.7.

#### 2.5.5. Parity Rule 2 Plot Analysis

The Parity Rule 2 (PR2) plot analysis evaluates codon usage bias by plotting GC3 bias (GC3 = G3/[G3 + C3]) on the horizontal axis against AU3 bias (AU3 = A3/[A3 + U3]) on the vertical axis, specifically examining the third codon positions of four-fold degenerate amino acids (Ala, Arg, Gly, Pro, Thr, and Val) because their third codon positions exhibit maximum degeneracy, making them particularly sensitive detectors of evolutionary constraints [27,28]. Bases with an even distribution imply a dominant role of mutational pressure; conversely, a deviation from this pattern indicates the influence of natural selection [29]. When data points cluster around the neutral equilibrium position (GC3 = AU3 = 0.5), this indicates mutation pressure as the dominant evolutionary force. Conversely, systematic deviations from this equilibrium reveal the combined influence of natural selection and mutational pressures [30]. In this research, the scatter plots were drawn using Python 3.13.7.

#### 2.5.6. Effective Number of Codons Analysis

The effective number of codons (ENc) is a metric used to quantify the absolute CUB by assessing the degree of CUB by coding sequences, independent of gene length and the number of amino acids [29]. The ENc value is calculated by the formula shown below [26]:

(5) $$\begin{array}{c} ENC=2+\frac{9}{F_{2}}+\frac{1}{F_{2}}+\frac{5}{F_{4}}+\frac{3}{F_{6}} \end{array}$$

where the F_k_ expression (k = 2, 3, 4, 6) indicates the values of F_k_ for k-fold degenerate amino acids. ENc values range from 20 (maximum CUB) to 61 (no CUB). When the ENc value of a codon is ≤35, it is considered a codon with significant CUB [31]. In this study, ENc values were computed using CodonW 1.4.2.

#### 2.5.7. ENc-GC3s Plot Analysis

An ENc-GC3s plot analysis is used to investigate the influence of GC3s content on codon usage. The ENc-GC3 plot is constructed with GC3 content on the x-axis, the observed effective number of codons (ENcobs) on the y-axis, displaying data points as a scatter plot, and the expected ENc values (ENcexp) as a standard curve. The ENcexp and ENcratio were calculated by the formula [32]:

(6) $$\begin{array}{c} ENcexp=2+GC3s+\frac{29}{{GC3s}^{2}+{\left(1-GC3s\right)}^{2}} \end{array}$$

(7) $$\begin{array}{c} ENcratio=\frac{ENcexp-ENcobs}{ENcexp} \end{array}$$

The ENcratio value quantifies the extent of variation between the expected and observed ENc values. In this research, the scatter plots were drawn using Python 3.13.7. If the data points cluster around this expected curve, it suggests that mutation pressure independently contributes to codon bias formation. If data points deviate significantly from the expected curve, it indicates the involvement of other factors, such as natural selection.

### 2.6. Nucleotide Diversity Analysis

The analysis of DNA polymorphisms is a powerful tool for understanding the evolutionary process and determining the functional significance of specific genomic regions. The sliding window technique was employed for exploratory analysis of DNA polymorphism data. Nucleotide diversity was evaluated using a sliding window analysis in DnaSP v5.10, with the following parameters: window size = 200 bp, step size = 20 bp [33], and the graph was generated using Python 3.13.7.

### 2.7. Positive Selective Analysis

To detect positive selection acting on mitochondrial protein-coding genes in the lineage leading to *Meiacanthus*, we employed the branch and branch-site models implemented in PAMLX 1.3.2 [34]. As *M. atrodorsalis*, *M. grammistes*, *M. mossambicus*, *M. tongaensis*, *A. taeniatus*, *P. rhinorhynchos, P. breviceps* and *X. setifer* are the only nemophine species with available mitogenomic data, we reconstructed the phylogenetic relationships among the eight species using RAxML v8.2.12, with *Omobranchus fasciolatoceps* as the outgroup and 1000 bootstrap replicates. The resulting tree was used for all subsequent codon-based analyses.

For each gene, the ratio of nonsynonymous to synonymous substitutions (dN/dS, ω) was estimated. The significance of positive selection was assessed using likelihood ratio tests (LRTs), comparing a null model (which constrains ω ≤ 1 on the foreground branch) against an alternative model that allows for ω > 1 on the foreground branch (the *Meiacanthus* lineage). For analyses using the branch-site model, individual codon sites with a posterior probability > 0.95 under the Bayes Empirical Bayes (BEB) method were considered to be under positive selection. Only genes for which the LRT was statistically significant (*p* < 0.05) were regarded as showing evidence of positive selection. The three-dimensional structure of proteins was predicted by SWISS-MODEL [35].

## 3. Results

### 3.1. The Structure of the Mitochondrial Genome Among Eight Nemophine Species

**Figure 1:**
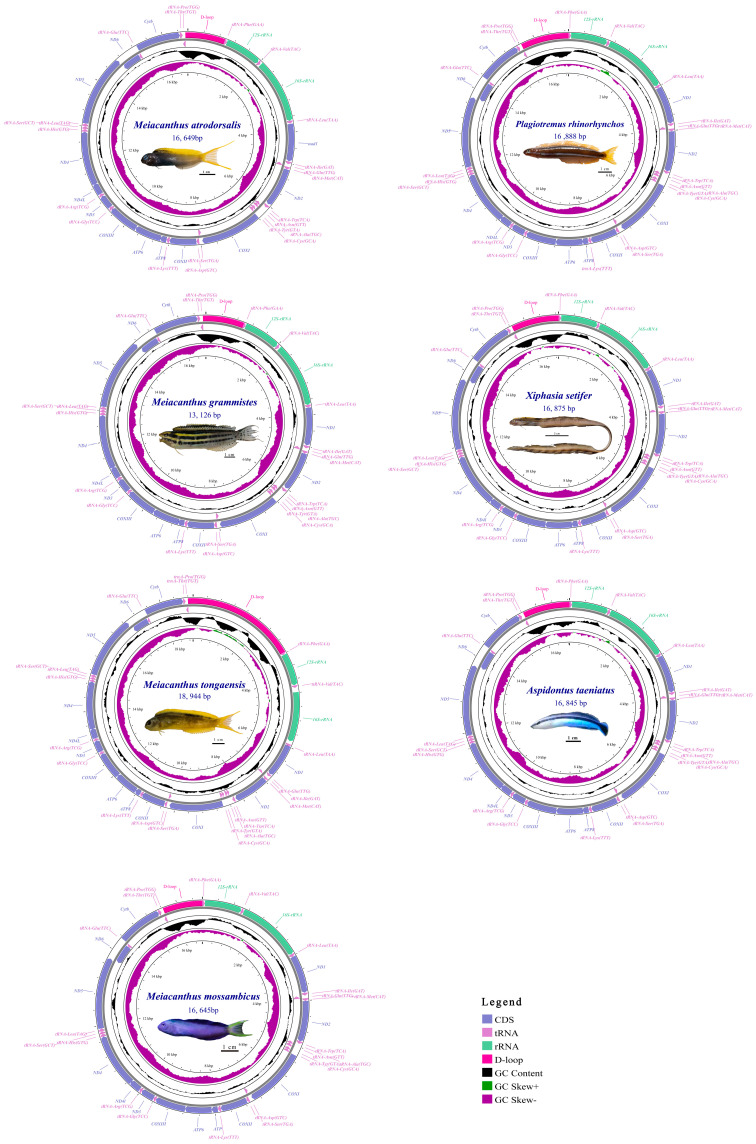
Mitogenome circular sketch of Nemophini provided in this study.

The circular maps of the mitochondrial genomes of eight nemophine species (*M. atrodorsalis*, *M. grammistes*, *M. tongaensis*, *M. mossambicus*, *A. taeniatus*, *P. rhinorhynchos*, *X. setifer* and *P. breviceps*) were shown in Figure 1. All genomes are closed circular molecules, each harboring 22 tRNA genes, 2 rRNA genes and 13 protein-coding sequences (CDS). No-coding regions were also found from these genomes. The mitochondrial gene arrangement was consistent with the typical gene order observed in ray-finned fishes (Supporting Information Figure S2). Among the 22 tRNA genes, *tRNA-Gln* (TTG), *tRNA-Ala* (TGC), *tRNA-Asn* (GTT), *tRNA-Cys* (GCA), *tRNA-Tyr* (GTA), *tRNA-Ser* (TGA), *tRNA-Glu* (TTC) and *tRNA-Pro* (TTC) are located on the light chain. Additionally, *ND6* is the only protein-coding gene that is located on the light chain.

### 3.2. Phylogeny and Ancestral State Reconstruction of Meiacanthus and Related Fishes

**Figure 2:**
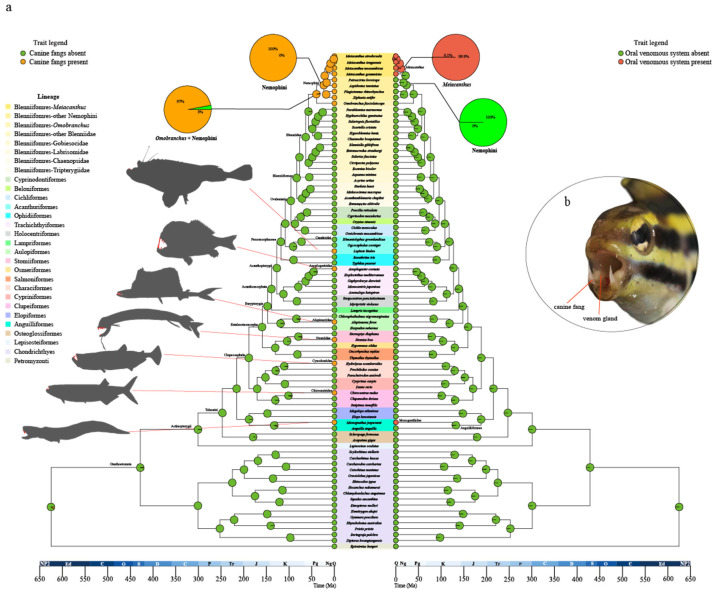
Evolution of canine fangs and the oral venom system in jawed fishes (Gnathostomata). (**a**) Phylogenetic tree of jawed fishes with ancestral state reconstructions for canine fangs (left) and the oral venom systems. Pie charts at the tree tips represent the presence or absence of enlarged canine fangs and the oral venom system respectively; pie charts at the internal nodes represent the marginal probability of each ancestral state of canine fangs and the oral venom system. (**b**) Close-up of the oral venom system in *M. grammistes*.

A phylogenetic tree encompassing 84 species of Gnathostomata was reconstructed, with the root age calibrated at 625 Ma (Figure 2). The results show that among all jawed fishes with enlarged canine fangs, *Monognathus* represents the earliest diverging lineage, splitting from other lineages approximately 132.56 million years ago. Our results support the division of Blenniidae into four major clades and confirm that Nemophini is a monophyletic group. *Meiacanthus* forms a monophyletic group with the (*Petroscirtes* + *Aspidontus*) clade. Additionally, our analyses indicate that Blenniidae originated around 63.04 Ma, the most recent common ancestor (MRCA) of *Omobranchus* and Nemophini lived approximately 39.11 Ma, and *Meiacanthus* originated around 11.31 Ma.

Ancestral state reconstruction reveals that the earliest jawed vertebrates lacked enlarged canine fangs and an oral venom system. Within our phylogenetic framework, enlarged canine fangs evolved independently at least nine times, with the earliest occurrence in *Monognathus* during the Early Cretaceous. In some lineages, only a few teeth were elongated (e.g., Chirocentridae and Nemophini), whereas in others, all teeth were elongated (e.g., Stomiidae). Blenniidae originated from a fangless ancestor, while the MRCA of Nemophini and *Omobranchus* was likely fang-bearing.

Within jawed fishes, the oral venom system evolved independently on two occasions: first in *Monognathus* and second in *Meiacanthus*. Although the MRCA of Nemophini likely possessed canine fangs, it probably did not have an oral venom system, whereas the MRCA of *Meiacanthus* was likely venomous. Therefore, during the evolution of *Meiacanthus*, the acquisition of canine fangs likely preceded the development of the oral venom system.

### 3.3. The Speciation Rate Is Higher in the Meiacanthus

**Figure 3:**
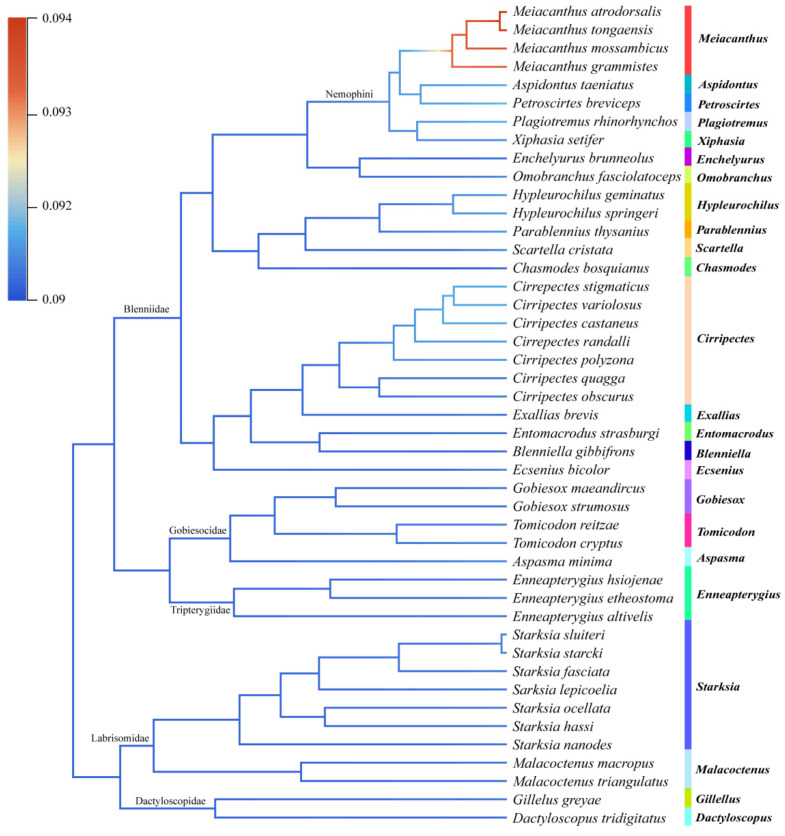
Speciation rate analysis based on the mitogenomic data of Blenniiformes. The speciation rate for each branch is shown in different colors.

A phylogenetic tree encompassing 45 Blenniiformes species was reconstructed for the speciation rate analysis. The speciation rate analysis indicates that *Meiacanthus* has experienced a higher speciation rate since diverging from other non-venomous nemophine blennies. The mean speciation rate (λ) for *Meiacanthus* (0.09392 ± 4.16 × 10^−5^) is 3.4% higher than the mean for the background lineages (0.09083 ± 2.35 × 10^−5^), suggesting an elevated speciation rate in this lineage (Figure 3).

### 3.4. The Nucleotide Composition Among Eight Nemophine Species

The GC content of each mitochondrial genome, along with the overall GC, GC1, GC2 and GC3 contents of their CDSs, as well as ENc values, are presented in Table 1. The overall GC content of mitochondrial genomes is comparable to those reported in other fish species, such as zebrafish and medaka [36,37]. The overall average GC content (43.02%) of mitochondrial genomes of *Meiacanthus* species is significantly higher than that of other nemophine species (40.25%, t = 3.93, *p* = 0.00775), indicating that *Meiacanthus* tends to use G/C nucleotides more frequently.

### 3.5. Codon Usage Bias Analysis for Nemophine Blennies

**Figure 4:**
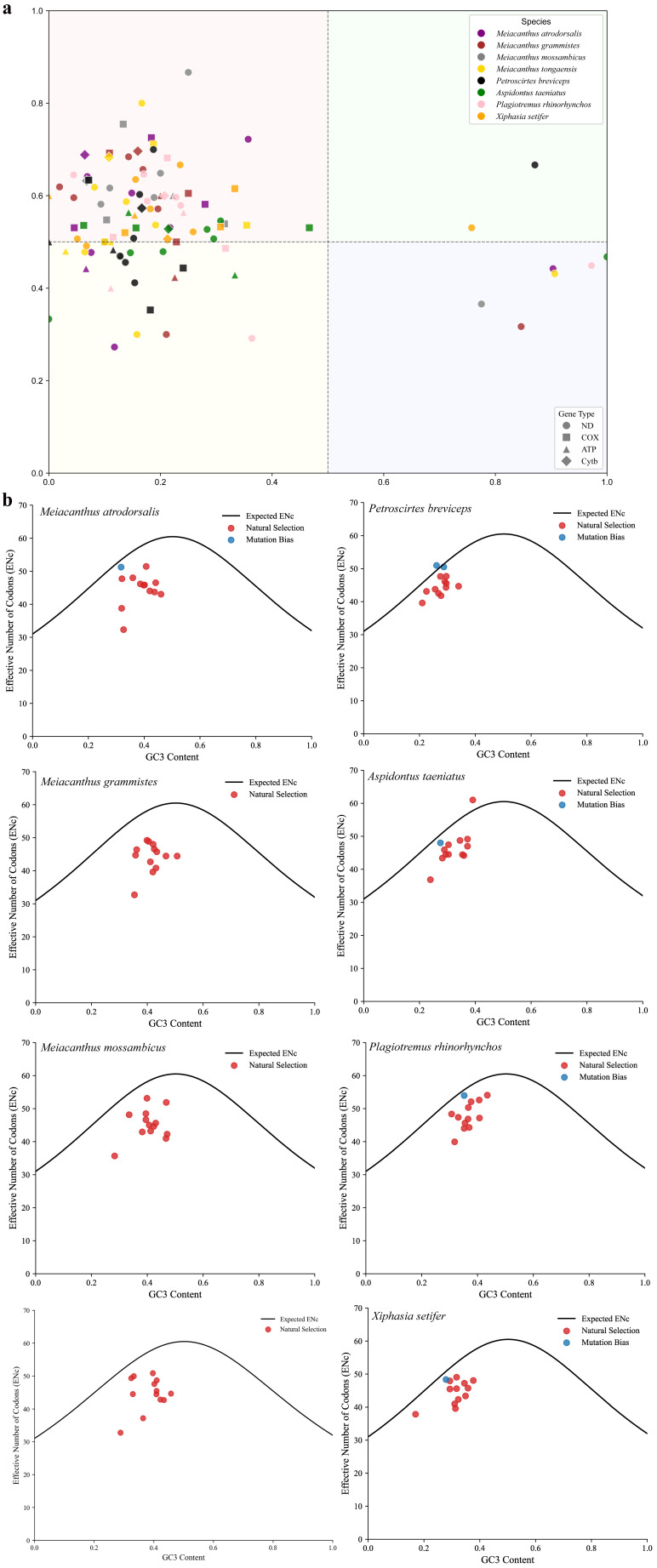
Codon usage bias analysis for nemophine species. (**a**) Parity Rule 2 (PR2) plot analysis of the third-codon position composition in nemophine blennies. Species are differentiated by color (see legend), while gene functional categories are indicated by point shapes. (**b**) ENc-GC3s plot analysis of third-codon position composition in nemophine blennies. Gene functional categories are differentiated by point shapes.

PR2-plot and ENc-GC3s plot analysis are common approaches for analyzing codon usage bias. Results of the PR2-plot analysis for nemophine species are presented in Figure 4a. In most species, the majority of codons fall within the fourth quadrant (AC, 53.8–84.6%), followed by the third quadrant (TC, 7.7–30.8%). The only exception is *Petroscirtes breviceps*, in which codons are predominantly distributed in the third quadrant (46.2%), followed by the fourth quadrant (38.5%). These findings indicate an asymmetric distribution of codon usage across quadrants, suggesting deviations from the equal use of complementary bases, and demonstrate that in addition to nucleotide mutation pressure, other factors also contribute to shaping codon usage bias in mitochondrial genomes.

The ENc-GC3s plot analysis results for each nemophine species are presented in Figure 4b. The scatter plots demonstrate high interspecific similarity, with all data points-except for *ND3* of *P. breviceps*-falling below the expected curve. Notably, all eight species exhibited absolute ENc ratio values exceeding 0.05 for their 13 protein-coding genes. These results indicate that codon usage bias in the mitochondrial genomes of Nemophini is influenced by natural selection, while that of the ND3 gene in *P. breviceps* is entirely determined by nucleotide mutation.

### 3.6. Relationship Between Codon Usage Bias and Gene Expression Among Nemophine Blennies

As shown in Table 2, all nemophine species exhibited significant positive correlations between CAI and CBI, with Pearson’s r-values ranging from 0.565 to 0.0446 (*p* < 0.05 for all species). These results demonstrate a consistent association between codon usage bias and mitochondrial gene expression across Nemophini.

### 3.7. Secondary Structure of Mitochondrial tRNA Among Eight Nemophine Species

**Figure 5:**
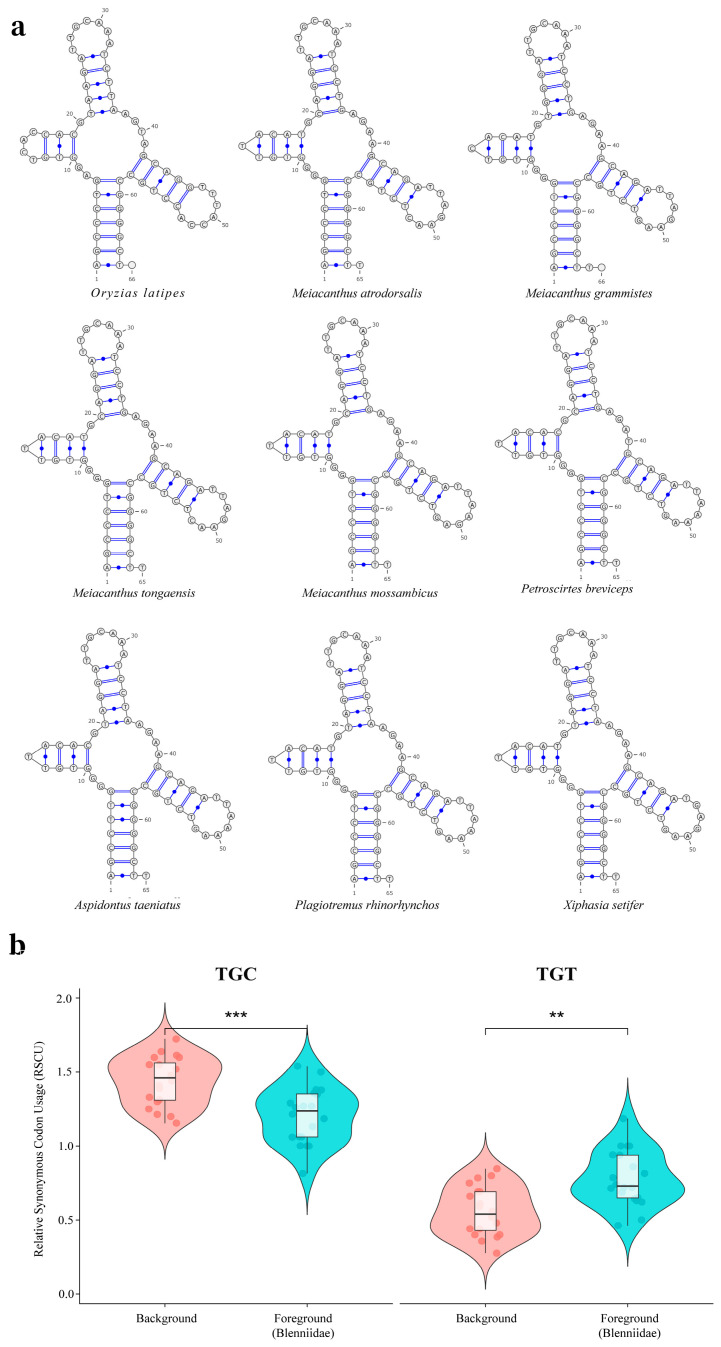
Comparative analysis of tRNA. (**a**) Secondary structure of a typical tRNA-Cys (GCA) with a complete cloverleaf structure which is detected from the medaka (*Oryzias latipes*) and tRNA-Cys (GCA) of 8 nemophine species in this study. (**b**) Violin plot of RSCU analysis for Blenniidae and fish species with a complete cloverleaf structure tRNA-Cys (designated as background). Asterisks indicate the level of statistical significance: ** *p* < 0.01, *** *p* < 0.001.

Mitochondrial tRNAs in nemophine blennies exhibit a high degree of structural conservation across species. Among the 22 tRNAs encoded in the mitochondrial genome, 20 conform to the canonical cloverleaf secondary structure. Consistent with other metazoans, nemophine species possess a tRNA-Ser (GCU) that completely lacks the DHU arm (both loop and stem). Notably, they also feature a tRNA-Cys (GCA) with only a single nucleotide in the DHU loop position (Figure 5a), a structural reduction rarely reported in other vertebrate mitochondrial genomes. After examining the data available from GenBank, we found that all Blenniidae fishes possess a similar tRNA-Cys (GCA), while other Ovalentaria fish such as cichlids retain the typical cloverleaf-shaped tRNA-Cys (GCA) [38].

To explore whether this unusual tRNA structure influences mitochondrial codon usage bias in blennies, we compared the relative synonymous codon usage (RSCU) values of the two cysteine codons (TGC and TGT) between blennid fishes and fishes possessing typical cloverleaf-structured tRNA-Cys. To minimize potential biases arising from phylogenetic distance, we restricted the comparison to species within Ovalentaria. Twenty blennid and twenty non-blennid Ovalentarian species were selected, with blennies assigned as the foreground group and non-blennid Ovalentarian species retaining canonical cloverleaf-structured tRNA-Cys (GCA) assigned as the background group. Violin plots were generated in R using the package ggplot2 v4.0.3 to visualize the distributions of RSCU values (Figure 5b). Compared with fishes possessing canonical cloverleaf-structured tRNA-Cys (GCA), blennid fishes exhibited significantly different Cys codon usage patterns. Although both groups showed a preference for the TGC codon, blennid fishes displayed a reduced bias toward TGC accompanied by a relative increase in TGT usage. Wilcoxon rank-sum tests revealed significant differences in the RSCU values of both Cys codons between the two groups (TGC: *p* = 0.0006; TGT: *p* = 0.0010).

### 3.8. Positive Selected in Most Protein-Coding Genes of Meiacanthus

**Figure 6:**
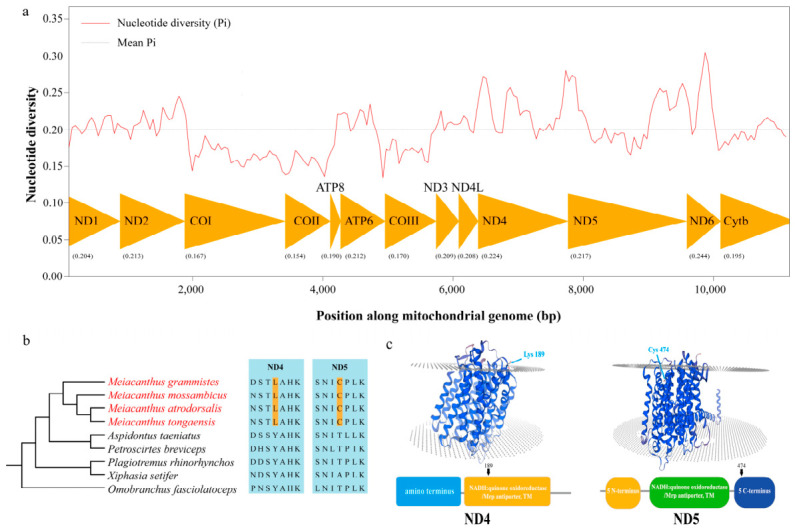
The result of positive selection and nucleotide diversity analysis. (**a**) Nucleotide diversities of 13 CDSs of *Meiacanthus*. (**b**) The phylogeny of nemophine species, showing the positive selective sites of ND4 and ND5, while species of *Meiacanthus* are highlighted in red. (**c**) The three-dimensional structures of ND4 and ND5 of *Meiacanthus* are shown on the right, the positive selective sites are pointed out by black arrows.

Sliding Window Analysis revealed relatively low nucleotide diversity across most of the 13 mitochondrial protein-coding genes (CDSs) of *Meiacanthus* (Figure 6a). Positive selection analyses identified significant selection signals in most mitochondrial protein-coding genes (*p* < 0.01), with the exception of *Cytb* (*p* = 0.14). Specifically, evidence of positive selection was detected in *ATP6*, *ATP8*, *COX1*, *COX2*, *COX3*, *ND1*, *ND2*, *ND3*, *ND4*, *ND4L*, *ND5*, and *ND6*.

Within these genes, two codons were identified as positively selected sites with high posterior probabilities (BEB > 0.95): (1) a tyrosine (Y) to leucine (L) substitution at position 189 of ND4; and (2) a threonine/alanine (T/A) to cysteine (C) substitution at position 474 of ND5 (Table 3, Figure 6b,c). We also noticed that the positive selective site of ND4 is located on the transmembrane (TM) domain of the NADH:quinone oxidoreductase/Mrp antiporter family, which may strengthen the efficiency of energy transducing. These nonsynonymous substitutions exhibited significantly stronger selection signals in the foreground (*Meiacanthus*) branches compared to background branches, indicating adaptive evolution in the mitochondrial genes of these venomous species.

Notably, despite the overall low nucleotide diversity of the *Meiacanthus* mitochondrial genome, sliding window analysis revealed that *ND4* and *ND5* display relatively higher nucleotide diversity (π) compared to other genes. This observation supports the adaptive signals detected in these two genes, suggesting they may be subject to ongoing evolutionary pressures.

## 4. Discussion

As the primary sites of cellular energy metabolism in eukaryotes, mitochondria are tightly linked to the energetic demands of organisms; thus, the characteristics of mitochondrial genomes can provide critical insights into lineage-specific adaptive evolution. Owing to their maternal inheritance and relatively high mutation rate, mitogenomes are also well-established markers for phylogenetic reconstruction and evolutionary inference. Given these properties, investigating the mitogenomes of *Meiacanthus* and its close relatives offers a unique opportunity to dissect the evolutionary processes underlying the origin and diversification of their oral venom system—a key adaptive trait in this lineage.

Our phylogenetic analyses confirm that Blenniidae can be divided into four major groups, support the monophyletic status of Nemophini, and verify its sister-group relationship with *Omobranchus* and *Enchelyurus*, both of which possess canine fangs, suggesting that canine fangs originated only once within Blenniidae. Importantly, our results place *Meiacanthus* as the sister group to the (*Aspidontus* + *Petroscirtes*) clade, which is consistent with Casewell et al. and Liu et al. [2,9]. This congruence strengthens confidence in the resolved phylogenetic framework and provides a reliable basis for subsequent evolutionary inference.

Divergence time estimates indicate that the most recent common ancestor (MRCA) of Blenniiformes emerged in the late Cretaceous, while Blenniidae originated and underwent rapid adaptive radiation shortly after the Cretaceous-Paleogene (K-Pg) extinction event. In contrast, *Meiacanthus,* the second-largest genus in Blenniidae, did not originate until the Late Miocene (~11.31 Ma). Despite its relatively short evolutionary history, *Meiacanthus* exhibits remarkably high species diversity, which aligns with the hypothesis proposed by Liu et al. [9] that the emergence of venom glands facilitated speciation in *Meiacanthus*.

Ancestral state reconstruction further clarifies the evolutionary sequence of key traits. The MRCA of Blenniidae probably lacked both enlarged canine fangs and an oral venom system, consistent with the algae-scraping diet of most blennies which is supported by their comb-like teeth. Enlarged canine fangs are likely to have first evolved in the common ancestors of Nemophini and *Omobranchus*, serving as an advantageous weapon for both offense and defense. Notably, the buccal venom gland appears to have arisen only with the origin of *Meiacanthus* in the late Miocene. Such an evolutionary sequence is different from that of snakes and aligns with the views of Bittenbinder et al. [39]. A remaining gap in our understanding concerns the evolutionary sequence of the oral venom system in *Monognathus*. As a highly specialized group, *Monognathus* possesses both canine fangs and an oral venom system—traits absent in all other anguilliform taxa. This extreme specialization complicates ancestral trait reconstruction, as there are no closely related non-venomous lineages to serve as comparative benchmarks. Resolving this ambiguity will require additional genomic data from *Monognathus* and its deep relatives.

The results of speciation rate analysis indicate that *Meiacanthus* experienced an elevated speciation rate following its divergence from its non-venomous sister group, which is consistent with previous findings [9], who proposed that the evolution of the oral venom system promoted rapid speciation within the genus. The oral venom system is a powerful weapon for self-defense, and confers great advantage for this lineage, which may explain the acceleration of speciation after the origin of the venom gland.

Analysis of codon usage bias can be applied to quantify the relative contributions of natural selection and neutral mutations to sequence variation. In the present study, we utilized PR2 plot and ENC–GC3s plot analyses, which are commonly employed to identify the primary factors shaping codon usage bias. The PR2 plot suggests that, in addition to mutational pressure, other factors also contribute to codon usage bias in nemophine mitochondrial genomes, while the ENC–GC3s plot indicates that at least part of these factors can be attributed to natural selection. Taken together, these results indicate that codon usage bias in nemophine mitochondrial genomes is influenced by both mutation and natural selection [40,41,42].

Previous studies have shown that gene expression is influenced by both CAI and CBI, and the correlation between these indices can quantify the link between CUB and translational efficiency [43,44]. In our analysis, all nemophine species showed significant correlations (*p* < 0.05). This strong association indicated that the CUB is strongly associated with gene expression in their mitochondrial genomes [40]. This finding suggests that codon usage preferences may play an important role in shaping translational efficiency and regulating mitochondrial gene function in Nemophini, providing new insights into the molecular mechanisms underlying mitochondrial genome evolution in this ecologically specialized lineage.

Mitochondrial tRNAs exhibit high structural conservation across metazoans, with the loss of the DHU (D) arm in tRNA-Ser(GCU) being a well-documented synapomorphy [45,46]. Our analysis uncovered a second unusual tRNA structure in Blenniidae: tRNA-Cys (GCA) with a truncated DHU loop (containing only a single nucleotide), which is a feature not previously reported in this lineage. D-arm-lacking tRNA-Cys (GCA) has been reported from vertebrates [47], yet the only example is the tuatara *Sphenodon punctatus*, which is phylogenetically distant from the group we focus on, and has lost the whole D-arm. Despite the fact that the presence of the D-arm can strengthen the stability of the RNA hairpin, it is reported that its absence does not substantially impair tRNA functionality. Studies confirm that mammalian D-arm-lacking tRNA-Ser (GCU) maintains comparable EF-Tu/GTP ternary complex formation capacity to canonical tRNA-Ser (UGA). In these animals, mitochondrial seryl-tRNA synthetase has evolved specialized helical domains to specifically recognize the acceptor stem of mt-tRNA-Ser (GCU), though the amount of peptide produced via this D-arm-lacking tRNA is less than using other tRNA-Ser. Due to the limited number of nucleotides remaining in the D-loop, which may be insufficient for seryl-tRNA synthetase binding, the tRNA-Cys (GCA) of Nemophini may also exhibit a similar situation. In this study, we found that the bias of TGC is reduced, which is the codon for tRNA-Cys (GCA), suggesting that the simplified tRNA-Cys (GCA) may reduce the translation efficiency of the TGC codon, resulting in an increased preference for TGT as a Cys codon as a translational compensatory mechanism.

In the sliding window analysis, we found that the nucleotide polymorphism of *ND4* and *ND5* was relatively high, which may be related to their role in the adaptive evolution of the *Meiacanthus*. All CDSs of *Meiacanthus*, except *Cytb*, exhibited signals of positive selection, and two positively selected sites were identified: one in the *ND4* gene and one in the *ND5* gene. Vertebrate mitochondrial CDSs are closely related to energy metabolism. *ND4* and *ND5*, as well as other *ND* genes, encode NADH dehydrogenase subunits of mitochondrial complex I, and are involved in the generation of ATP through the respiratory chain. The positive selection of *ND* genes has been reported in numerous species, and is usually explained to be connected to metabolic efficiency [48,49,50,51,52]. The positive selection of *COX* and *ATP* genes is also found in lineages with high metabolic demand [53,54]. We therefore hypothesize that the observed positive selection in *Meiacanthus* may be associated with venom gland development, as toxin synthesis demands considerable energy expenditure. However, we still lack direct evidence regarding whether positive selection on *ND4* and *ND5* drives venom biosynthesis, so further experiments are needed to validate this link.

As the only known fish in coral reef ecosystems possessing an oral venom system, the genus *Meiacanthus* exhibits specific adaptive characteristics. These adaptations have greatly enhanced the fitness of *Meiacanthus*, making it the largest genus of Nemophini and the second-largest genus in Blenniidae within just 11 million years. Despite nearly 200 years having passed since *Meiacanthus* species were first scientifically described, and decades of their circulation as ornamental fishes, research on them has long been limited to the relatively superficial aspects of their mimicry systems and the composition and effect of their venom for a long time. This study is the first to investigate *Meiacanthus* and its close relatives at the level of complete mitochondrial genomes, elucidating the evolutionary history of their oral venom system, and providing a new perspective on the origin and development of the oral venom system in vertebrates. However, this study relies exclusively on computational analyses of mitogenomic data, and our conclusions currently lack validation from functional assays, such as transcriptomic and proteomic data. Future studies should prioritize validating the inferences and hypotheses proposed herein from this functional perspective. Furthermore, given that mitochondrial genomes encode only a limited suite of genes, the scope of information and functional insight they can provide is inherently restricted. The majority of genes relevant to the venom system likely reside within the expansive nuclear genome and may play more direct and pivotal roles in the development and functioning of the oral venom apparatus. Mining these nuclear-encoded genes will be essential for exploring the specific molecular mechanisms underlying the evolution of the oral venom system in *Meiacanthus*.

## 5. Conclusions

This study represents the first comprehensive analysis of *Meiacanthus* and its relatives based on complete mitogenomes, clarifying the phylogenetic position and evolutionary history of *Meiacanthus*. While our mitogenomic data highlight energetic adaptations supporting venom synthesis, the nuclear genes encoding venom toxins and their regulatory pathways remain uncharacterized. Future studies integrating nuclear genomic data with transcriptomic analyses of venom glands will be essential to fully dissect the genetic basis of venom evolution in this unique lineage, and to contextualize its adaptive significance within the broader framework of vertebrate venom system convergence.

## Figures and Tables

**Table 1:** GC content of mitochondrial genomes and GC composition of CDSs, together with ENc value.

	Whole Genome GC	CDS GC	GC1	GC2	GC3	ENc
*M. atrodorsalis*	42.42%	39.1%	42.83%	45.14%	42.31%	47.18
*M. grammistes*	43.02%	44.7%	49.84%	40.76%	42.38%	46.60
*M. tongaensis*	43.60%	44.6%	49.24%	40.90%	39.64%	53.79
*M. mossambicus*	43.05%	44.5%	49.93%	40.91%	41.59%	47.17
*P. breviceps*	40.40%,	41.3%	49.54%	41.09%	32.42%	47.77
*A. taeniatus*	38.49%	39.1%	48.56%	40.63%	27.52%	45.68
*P. rhinorhynchos*	41.72%	43.1%	50.73%	41.23%	36.75%	48.67
*X. setifer*	40.38%	41.2%	48.93%	41.15%	32.83%	47.10

**Table 2:** Correlation analysis between CAI and CBI.

	r	*p*
*M. atrodorsalis*	0.6275	0.0217 *
*M. grammistes*	0.7901	0.0013 *
*M. tongaensis*	0.5641	0.0446 *
*M. mossambicus*	0.5980	0.0309 *
*P. breviceps*	0.7028	0.0017 **
*A. taeniatus*	0.6796	0.0106 *
*P. rhinorhynchos*	0.7351	0.0042 **
*X. setifer*	0.6656	0.0130 *

CAI, Codon Adaptation Index; CBI, Codon Bias Index. The numbers in the table represent the Pearson correlation coefficient (r) and its probability value (*p*), where asterisks indicate the level of statistical significance: * *p* < 0.05, ** *p* < 0.01.

**Table 3:** Selective pressure analyses of the mitochondrial genes of *Meiacanthus*.

Gene	Model	lnL	Estimates of Parameter					Model Compared	2lnL	LRT *p*-Value	Positive Site
*ND4*	Model A	−734.751394	Branch site model					Model A vs. Model A null	142.413	0	189,L,0.950 *
			site class	0	1	2a	2b				
			proportion	0.94155	0.04545	0.01241	0.0006				
			background ω	0.02054	1	0.02054	1				
			Foreground ω	0.02054	1	233.18938	233.18938				
	Model A null	−6805.957907									
*ND5*	Model A	−8676.049383	Branch site model					Model A vs. Model A null	177.947	0	474,C,0.950 *
			site class	0	1	2a	2b				
			proportion	0.94424	0.04765	0.00772	0.00039				
			background ω	0.01887	1	0.01887	1				
			foreground ω	0.01887	1	3.81482	3.81482				
	Model A null	−8765.022698									

* Asterisks indicate positively selected sites identified by the Bayes Empirical Bayes (BEB) method with posterior probability ≥ 0.95.

## Data Availability

The mitogenomic sequence data that support the findings of this study are openly available in the GenBank of NCBI at https://www.ncbi.nlm.nih.gov/, the accessions of each sequence of *Meiacanthus atrodorsalis*, *Meiacanthus grammistes*, *Meiacanthus tongaensis*, *Meiacanthus mossambicus*, *Plagiotremus rhinorhynchos*, *Aspidontus taeniatus*, *Xiphasia setifer* are PX126059, PX207684, PX207683, PX207685, PX127636, PX206071 and PX170677 respectively.

## Supplementary Materials

The following supporting information can be downloaded at https://www.mdpi.com/article/10.3390/ani16152315/s1, Figure S1: The sequencing depth and coverage map of the complete mitochondrial genome sequence of nemophine blennies provided by this study. The x- and y-axes represent genomic position and sequencing depth respectively; Figure S2: The mitochondrial gene arrangement of nemophine blennies and other ray-finned fishes. The green, purple, and red arrows indicate tRNA genes, rRNA genes, and protein-coding genes, respectively; Table S1: List of GenBank accession numbers of all 84 species used for phylogenetic analysis; Table S2: List of GenBank accession numbers and references of all 45 species used for speciation rate analysis.
