# Supplementary material for: Comparative Mitogenomics of Meiacanthus Blennies Reveals Venom System Evolution and Adaptive Traits
Source: Animals (Basel). 2026 Jul 27;16(15):2315. doi: 10.3390/ani16152315 (PMC13463496; doi:10.3390/ani16152315)
Supplement: Supplementary file 1 [file animals-16-02315-s001.zip › Supporting Information Figure S1.pdf]

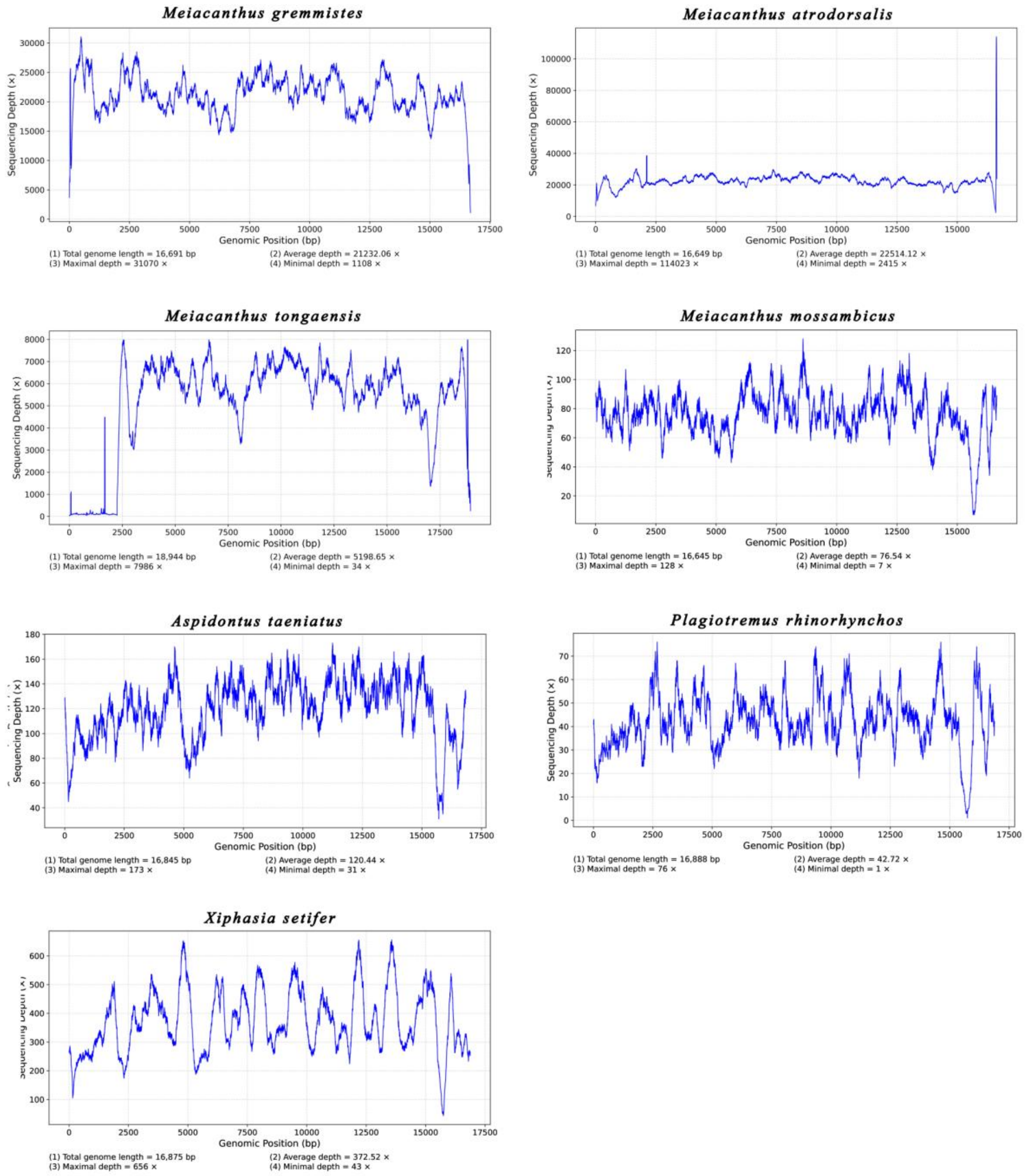

Figure S1. The sequencing depth and coverage map of the complete mitochondrial genome sequence of nemophine blennies provided by this study. The x- and y-axes represent genomic position and sequencing depth respectively.
