# Supplementary material for: Comparative Mitogenomics of Meiacanthus Blennies Reveals Venom System Evolution and Adaptive Traits
Source: Animals (Basel). 2026 Jul 27;16(15):2315. doi: 10.3390/ani16152315 (PMC13463496; doi:10.3390/ani16152315)
Supplement: Supplementary file 1 [file animals-16-02315-s001.zip › Supporting Information Figure S2.pdf]

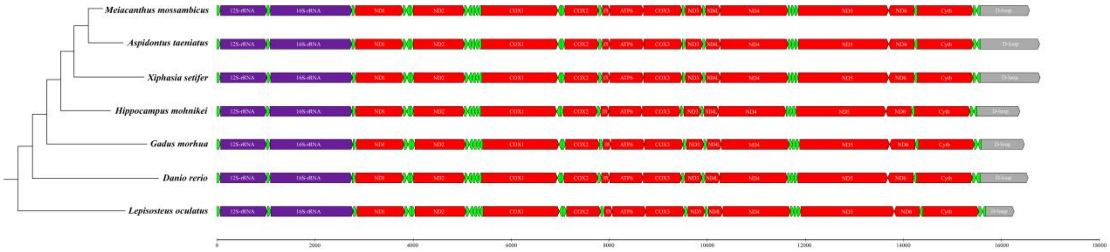

Figure S2. The mitochondrial gene arrangement of nemophine blennies and other ray-finned fishes. The green, purple, and red arrows indicate tRNA genes, rRNA genes, and protein-coding genes, respectively.
