# Supplementary material for: Comparative Mitogenomics of Meiacanthus Blennies Reveals Venom System Evolution and Adaptive Traits
Source: Animals (Basel). 2026 Jul 27;16(15):2315. doi: 10.3390/ani16152315 (PMC13463496; doi:10.3390/ani16152315)
Supplement: Supplementary file 1 [file animals-16-02315-s001.zip › Supporting Information Table S1.pdf]

Table S1. List of GenBank accession numbers of all 84 species used for phylogenetic analysis.

Spceies	Acession
Acanthemblemaria chaplini	PV460840.1
Acyrtus artius	PV460856.1
Alepisaurus ferox	NC_067732.1
Anguilla anguilla	KJ564270.1
Anomalops katoptron	NC_008128.1
Anoplogaster cornuta	NC_004391.1
Arapaima gigas	NC_010570.1
Barathrites iris	PV498538.1
Aspasma minima	NC_008130.1
Aspidontus taeniatus	PX206071
Beringraja pulchra	NC_025498.1
Blenniella gibbifrons	OR546230.1
Carcharhinus leucas	NC_023522.1
Carcharodon carcharias	NC_022415.1
Cetorhinus maximus	NC_023266.1
Chasmodes bosquianus	OR546170.1
Chirocentrus nudus	AP018761.1
Chlamydoselachus anguineus	NC_022729.1
Chlorophthalmus nigromarginatus	NC_027654.1
Choerodon azurio	NC_086888.1

Cichla monoculus	NC_084243.1
Cirripectes polyzona	NC_083064.1
Clupanodon thrissa	NC_018600.1
Cyprinodon macularius	NC_056955.1
Cyprinus carpio	KP993139.1
Danio rerio	NC_002333.2
Dipturus kwangtungensis	KF318309.2
Ecsenius bicolor	NC_028295.1
Elops hawaiensis	NC_005798.1
Enneanectes altivelis	MZ365315.1
Entomacrodus strasburgi	NC_083017.1
Eptatretus burgeri	NC_002807.1
Etmopterus molleri	LC649806.1
Gephyroberyx darwinii	NC_082998.1
Gymnura poecilura	NC_024102.1
Harpadon nehereus	MH204885.1
Hemitrygon akajei	NC_021132.1
Hexanchus nakamurai	NC_022733.1
Himantolophus groenlandicus	NC_013868.1
Hoplostethus mediterraneus	PV339945.1
Hydrolycus scomberoides	AP011989.1
Hypleurochilus geminatus	OP035308.1

Hypomesus olidus	NC_026566.1
Hypsoblennius hentz	OP056806.1
Lampris incognitus	NC_081041.1
Lepisosteus oculatus	AY442350.1
Lophius litulon	NC_023828.1
Malacoctenus macropus	OP056938.1
Megalops atlanticus	AP004808.1
Meiacanthus atrodorsalis	PX126059
Meiacanthus grammistes	PX207684
Meiacanthus tongaensis	PX207683
Meiacanthus mossambicus	PX207685
Monocentris japonicus	NC_004392.1
Monognathus jesperseni	AP010869.1
Myripristis violacea	NC_085254.1
Ogcocephalus corniger	PV660499.1
Omobranchus fasciolatoceps	OM677696.1
Oncorhynchus mykiss	L29771.1
Orectolobus japonicus	KF111729.1
Oreochromis mossambicus	PV820012.1
Oryzias sinensis	NC_013434.1
Parablennius marmoreus	OP035122.2
Paracheirodon axelrodi	AB898197.1

Petroscirtes breviceps	NC_028157.1
Plagiotremus rhinorhynchos	PX127636
Poecilia reticulata	NC_024238.1
Pristis pristis	NC_039438.1
Prochilodus costatus	NC_027690.1
Rhincodon typus	NC_023455.1
Rhincodon typus	ON065567.1
Salarias fasciatus	AP004451.1
Salariopsis fluviatilis	PP928772.1
Sargocentron punctatissimum	NC_085258.1
Scartella cristata	NC_085750.1
Scleropages formosus	NC_007012.1
Scyliorhinus stellaris	NC_066805.1
Setipinna tenuifilis	NC_051981.1
Squalus acanthias	NC_002012.1
Starksia hassi	PV460834.1
Sternoptyx diaphana	NC_051534.1
Stomias boa	PV704610.1
Thymallus thymallus	OR537182.1
Typhlias pearsei	NC_061376.1
Xiphasia setifer	PX170677
