# Supplementary material for: Comparative Mitogenomics of Meiacanthus Blennies Reveals Venom System Evolution and Adaptive Traits
Source: Animals (Basel). 2026 Jul 27;16(15):2315. doi: 10.3390/ani16152315 (PMC13463496; doi:10.3390/ani16152315)
Supplement: Supplementary file 1 [file animals-16-02315-s001.zip › Supporting Information Table S2.pdf]

Table S2. List of GenBank accession numbers and references of all 45 species used for speciation rate analysis.

Spceies	Acession
Aspasma minima	NC_008130.1
Aspidontus taeniatus	PX206071
Blenniella gibbifrons	OR546230.1
Chasmodes bosquianus	NC_083021.1
Cirripectes castaneus	NC_073148.1
Cirripectes obscurus	OP035135.2
Cirripectes polyzona	NC_083064.1
Cirripectes quagga	OP035151.1
Cirripectes randalli	NC_073155.1
Cirripectes stigmaticus	NC_073503.1
Cirripectes variolosus	OP035123.2
Dactyloscopus tridigitatus	OP056985.1
Ecsenius bicolor	NC_028295.1
Enchelyurus brunneolus	PV370339.1
Enneanectes altivelis	MZ365315.1
Enneapterygius etheostoma	AP006025.1
Enneapterygius hsiojenae	NC_080985.1
Entomacrodus strasburgi	NC_083017.1
Exallias brevis	OP035104.1
Gillellus greyae	OP056843.1

Gobiesox maeandricus	PX740303.1
Gobiesox strumosus	PX740318.1
Hypleurochilus geminatus	PX940017.1
Hypleurochilus springeri	NC_083056.1
Malacoctenus macropus	OP056938.1
Malacoctenus triangulatus	OP056825.2
Meiacanthus atrodorsalis	PX126059
Meiacanthus grammistes	PX207684
Meiacanthus tongaensis	PX207683
Meiacanthus mossambicus	PX207685
Omobranchus fasciolatoceps	OM677696.1
Parablennius thysanius	OP035092.1
Petroscirtes breviceps	NC_028157.1
Plagiotremus rhinorhynchos	PX127636
Starksia fasciata	OP056981.1
Scartella cristata	NC_085750.1
Starksia hassi	PV460834.1
Starksia lepicoelia	OP056836.1
Starksia nanodes	NC_083059.1
Starksia ocellata	OP056943.2
Starksia sluiteri	OP056854.1
Starksia starcki	OP056949.1

Tomicodon cryptus	NC_083053.1
Tomicodon reitzae	OP056862.1
Xiphasia setifer	PX170677
